# Pseudogout at the knee joint will frequently occur after hip fracture and lead to the knee pain in the early postoperative period

**DOI:** 10.1186/s13018-014-0145-9

**Published:** 2015-01-14

**Authors:** Kengo Harato, Hiroki Yoshida

**Affiliations:** Department of Orthopedic Surgery, Kawasaki Municipal Kawasaki Hospital, 12-1, Shinkawadouri, Kawasaki-ku, Kawasaki City, Kanagawa 210-0013 Japan; Department of Orthopedic Surgery, National Hospital Organization, Tochigi National Hospital, 1-10-37, Nakatomatsuri, Utsunomiya City, Tochigi 320-8580 Japan; Department of Orthopedic Surgery, Keio University School of Medicine, 35 Shinanomachi, Shinjuku, Tokyo, 160-8582 Japan

## Abstract

**Background:**

Symptomatic knee joint effusion is frequently observed after hip fracture, which may lead to postoperative knee pain during rehabilitation after hip fracture surgery. However, unfortunately, very little has been reported on this phenomenon in the literature. The purpose of the current study was to investigate the relationship between symptomatic knee effusion and postoperative knee pain and to clarify the reason of the effusion accompanied by hip fracture.

**Methods:**

A total of 100 patients over 65 years of age with an acute hip fracture after fall were prospectively followed up. Knee effusion was assessed on admission and at the operating room before the surgery. If knee effusion was observed at the time of the surgery, synovial fluid was collected into syringes to investigate the cause of the effusion using a compensated polarized light microscope. Furthermore, for each patient, we evaluated age, sex, radiographic knee osteoarthritis (OA), type of the fracture, laterality, severity of the fracture, and postoperative knee pain during rehabilitation. These factors were compared between patients with and without knee effusion at the time of the surgery. As a statistical analysis, we used Mann–Whitney U-test for patients’ age and categorical variables were analyzed by chi-square test or Fisher’s exact test.

**Results:**

A total of 30 patients presented symptomatic knee effusion at the time of the surgery. In patients with knee effusion, numbers of intertrochanteric fracture, radiographic knee OA, and postoperative knee pain were significantly large compared to those without effusion. In terms of synovial fluid analysis, calcium pyrophosphate dihydrate crystals were observed in 80% of patients with knee effusion.

**Conclusion:**

From our study, approximately 63% of patients with knee effusion at the time of the surgery had postoperative knee pain. In addition, this effusion was basically related to pseudogout.

## Introduction

Hip fracture is a major cause of impairment and disability among the elderly people [1]. The assessment of osteoporotic fracture risk in a patient requires the consideration of a number of different risk factors, including age, sex, bone mineral density, and previous fracture, in addition to a number of other clinical risk factors [2]. Currently, Japan has the world’s highest longevity rate, and the percentage of elderly people (older than 65 years) is expected to increase in the future. In addition, the number of hip fracture surgery may also increase during next decade.

Although symptomatic knee joint effusion is frequently observed after hip fracture surgery, very little has been reported on this phenomenon on the literature [3-5]. This effusion at the knee joint could lead to knee pain after hip fracture surgery. However, the correlation between knee effusion and postoperative knee pain has not been evaluated so far. Knee pain is one of the important factors that deteriorate the postoperative walking ability after hip fracture [6]. Furthermore, in recent studies, knee pain is one of the risk factors for falling [7,8]. In fact, during postoperative rehabilitation, patients frequently present as knee joint effusion and pain, which impairs the ability to walk. The reasons of this knee pain are still unknown.

It was hypothesized that knee effusion should be related to postoperative knee pain after hip fracture. The purpose of the current study was to concentrate on the knee condition after acute hip fracture from admission to discharge and to clarify the reason of the effusion.

## Materials and methods

### Participants

From December 2007 to March 2009, 140 patients, over the age of 65 years, with an acute hip fracture after fall were prospectively followed up. Patients were averaged 82 (65–102) years old. Patients who were too demented to answer the question given by clinicians were excluded from our study (25 patients). Patients with rheumatoid arthritis, any previous surgical history around the knee, and preoperative subjective knee pain were also excluded (three patients). Further, Patients who could not walk before injury were also excluded (12 patients). All the patients underwent the surgery as soon as possible after admission. Intramedullay nail or compression hip screw was selected for intertrochanteric fracture. Hemiarthroplasty or cannulated cancellous hip screw was chosen for subcapital fracture. Patients underwent a standard rehabilitation program which consisted of early range of motion and weight-bearing exercises as tolerated. All patients received prophylactic antibiotics and oral pain killer as necessary. All the subjects provided informed consent and the study was approved by our institution. Patients were followed for a period of 33–101 (mean 53) days.

### Data evaluations

**Figure 1:**
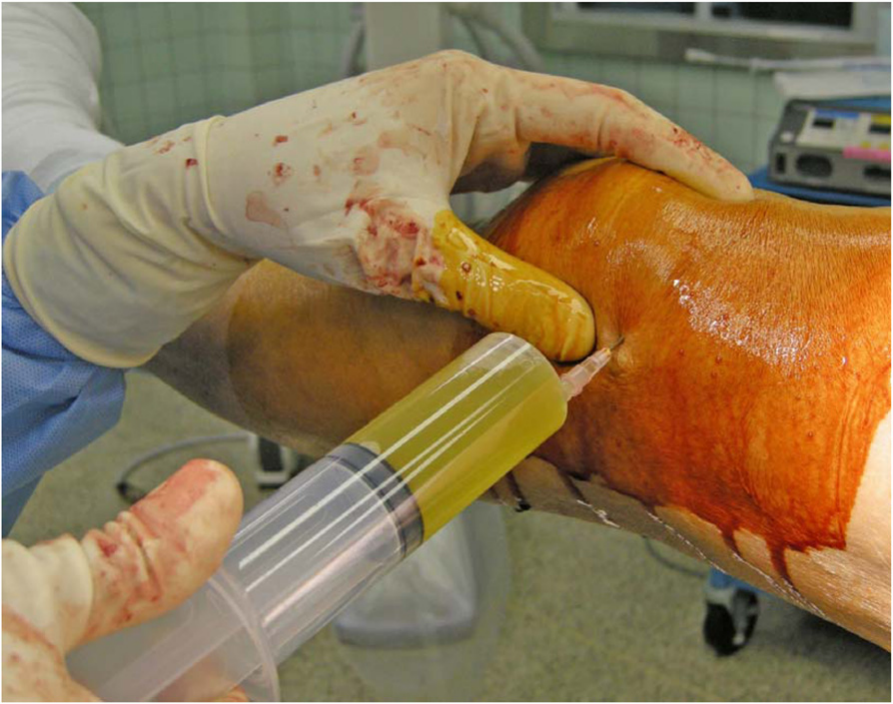
**Synovial fluid was collected into syringes at the time of the surgery.** Basically, a turbid synovial fluid was obtained.

Knee effusion was assessed on admission and at the operating room before the surgery. In addition, we evaluated age, sex, radiographic knee osteoarthritis (OA), calcification in meniscus or cartilage on plain radiographs, type of the fracture (intertrochanteric or subcapital), laterality (right or left), severity of the fracture (unstable or stable), and postoperative knee pain in first walking. Weight-bearing knee radiograph was taken in each patient on postoperative day 7. We characterized a knee as having radiographic OA if it had a Kellgren/Lawrence grade of ≥2 [9] and as having subjective pain if the patient had a visual analogue scale (VAS: 0–100 mm) of ≥10 mm when the patient could start first ambulation. In terms of the fracture severity (stable or unstable), Evans or Garden grade was used to determine the stability in intertrochanteric or subcapital fracture, respectively. In patients with knee effusion, synovial fluid was collected into syringes at the operating room immediately after the surgery (Figure 1), and one drop of each synovial fluid was analyzed for calcium pyrophosphate dihydrate crystals (CPPD) using a compensated polarized light microscope. Finally, we evaluated patients’ subjective knee pain again using VAS at the latest follow-up time.

### Statistical analysis

First, all the data, including age, sex, laterality, type of fracture, fracture severity, knee OA, calcification, and knee pain, were compared between patients with and without knee effusion using Mann–Whitney U-test or chi-square test (Fisher’s exact test). Second, we performed multiple logistic regression analysis to identify the important factor affecting the knee effusion at the time of the surgery. Knee effusion was treated as a dependent variable, and age, sex, laterality, type of fracture, fracture severity, and knee OA were treated as independent variables. *P* values of <0.05 were considered as significant. All statistical analyses were done with use of SPSS® Version 11 for Microsoft Windows (Chicago, IL).

## Results

### Patient demographics in each group

A total of 100 patients met our inclusion criteria. At the admission time, knee effusion was not found in each patient. Mean operative delay was 6 days (1–15). All the patients could walk with or without supportive tool preoperatively. Twenty-eight patients had knee pain when the patient could start first ambulation. Therefore, relatively high incidence of postoperative knee pain was observed. Interestingly, postoperative knee pain was observed in ipsilateral side (hip fracture side), except for only one case. Thirty patients had knee effusion (7–45 ml, mean 25 ml) at the time of the hip surgery although little effusion was found at admission. Nineteen of 30 patients (approximately 63%) with knee effusion had subjective knee pain in first ambulation. Synovial fluid obtained at the time of the hip fracture surgery was usually turbid (Figure 1). In addition, knee effusion was always found in ipsilateral side. At the latest follow-up time, eight patients had subjective knee pain. Specifically, knee pain improved during postoperative rehabilitation in 20 of 28 patients.

### Comparison between with and without knee effusion (Table 1)

**Table 1:** **Each evaluation was compared between patients with and without knee effusion at the operating room before the surgery**

**Evaluation**	**Effusion + (** ***N*** **= 30)**	**Effusion − (** ***N*** **= 70)**	***P*** **value** ^**a**^
Age	84.1 ± 8.0	81.2 ± 8.0	0.08
Sex (female/male)	24/6	50/20	0.52
Laterality (left/right)	18/12	43/27	0.89
Type (intertrochanteric/subcapital)	23/7	37/33	0.04*
Severity (unstable/stable)	24/6	58/12	0.95
Radiographic knee OA (+/−)	21/9	21/49	0.00047*
Calcification on plain radiograph (+/−)	22/8	2/68	<0.00001*
Knee pain in the first gait (+/−)	19/11	9/61	<0.00001*
Knee pain at the latest follow-up (+/−)	6/24	2/68	0.0085*

*Statistical significance (*p* < 0.05).

^a^Values obtained using Mann–Whitney U-test or chi-square test (Fisher’s exact test).

Results of comparison between with and without knee effusion are seen in Table 1. In effusion group, patients were relatively older and the number of intertrochanteric fracture was significantly large. In addition, significant differences were seen in knee OA, calcification, and subjective knee pain between groups. Specifically, subjective knee pain in first gait and at the latest follow-up time notably occurred in effusion group.

### Factors affecting the knee effusion (Table 2)

**Table 2:** **Detailed information of the multiple logistic regression analysis**

	**Partial regression coefficient**	***P***	**Odds ratio**	**95% confidence interval**
Intertrochanteric fracture	1.15	<0.05	3.2	1.1–9.0
Knee	1.69	<0.01	5.5	2.0–14.9

Model chi-square *p* < 0.01. Predictive accuracy 81.9%.

Results of multiple logistic regression analysis are shown in Table 2. The analysis in the current study would show the appropriate results, as predictive accuracy was 81.9%. According to the results, type of the hip fracture and radiographic knee OA were selected, while other factors including age, sex, laterality, and severity of the fracture were not selected. Odds ratios of type of the intertrochanteric fracture and radiographic knee OA were 3.2 and 5.5, respectively.

### Synovial fluid study at the time of the surgery

Synovial fluid aspiration was performed in 30 cases, and latter 15 cases of the patients were selected for analysis of CPPD, as we did not notice the existence of CPPD in the first 15 cases. In terms of synovial fluid study, CPPD was seen in 12 of 15 (80%) patients. On the other hand, no crystals were detected in the rest three cases.

## Discussion

Our results support our hypothesis that radiographic knee osteoarthritis would be related to the postoperative knee pain after hip fracture. In the present study, knee effusion was frequently found at the time of the surgery (30 patients) as previously described [3-5], and this phenomenon was basically related to CPPD (80%). Calcium pyrophosphate dihydrate deposition disease is known as pseudogout which is usually characterized by an inflammatory arthritis [10,11]. Acute pseudogout attacks present as joint pain, large joint effusions, and microscopic CPPD crystals in the synovial fluid. Acute attacks are basically self-limiting, lasting on average of 10 days even if the disease is left untreated [12]. After recurrent attacks, chronic arthritis with symptoms mimicking degenerative osteoarthritis may occur. In previous studies, calcium-containing crystals, including CPPD and basic calcium phosphate crystals are found in 60% of osteoarthritic synovial fluids obtained from patients with end-stage OA [13]. Our results suggested that postoperative knee pain could be basically caused by CPPD. In the current study, knee pain was self-limiting in 20 of 28 patients (71.4%) by the latest follow-up time. However, eight patients still had knee pain at that time though the true reason of the subjective knee pain was unknown. Presumably, this pain seemed to be related to osteoarthritis or pseudogout, as significantly large number of patients had knee pain in the effusion group. Therefore, if knee effusion is observed at the time of the surgery, appropriate treatment should be considered for the prevention of knee pain during rehabilitation in the early postoperative period.

Interestingly, subjective knee pain and effusion were found in ipsilateral side. If compensation mechanism against hip fracture exists, knee pain during rehabilitation should be observed in contralateral side because of greater loading on the unaffected side. Though hip fracture can sometimes cause referred pain at the knee joint, referred pain is never related to knee effusion, and thus, we believe that subjective knee pain in the current study is correlated to the knee joint itself. According to previous reports, ipsilateral knee effusion after hip surgery would be correlated with abnormal stress to the knee joint at the time of the injury or the operation [3]. However, in the present study, knee effusion was confirmed at the operating room before the surgery. Although this reason is still unknown, it is possible that abnormal stress at the ipsilateral knee joint may cause pseudogout attacks during waiting period until hip fracture surgery, as little effusion was found on admission.

Second hip fractures are a serious problem for the elderly people after hip fracture surgery, and the incidence was reported to be approximately 10% [14-16]. Generally, subjective knee pain was a risk factor for multiple falls in women [17]. Thus, prevention of the knee pain is an important issue for patients after hip fracture surgery. If patients with radiographic knee OA had an acute intertrochanteric fracture, careful attention should be taken for postoperative knee pain.

Potential limitations should be noted in the current study. First, the current study was conducted at our institution (Tochigi Prefecture, Japan). Our institution is located at rural districts consisted of super-aged population. Therefore, the current study may not be representative of the results obtained in other locations or countries. Second, relatively small samples were selected and follow-up was short in the present study, as knee condition was assessed in detail. Third, follow-up periods varied with a wide range in the present study. Therefore, these differences could influence the postoperative clinical evaluation. However, as our prospective study was the first to examine pre- and postoperative knee condition, our results offer useful information when considering factors affecting the postoperative knee pain following hip fracture surgery.

In conclusion, from our study, subjective knee pain during gait in the early postoperative period was related to knee effusion at the time of the surgery. In addition, this effusion after acute hip fracture was considered to be pseudogout.
